# Pleiotropic Associations with Alzheimer’s Disease and Physical Activity: Sex Differences and the Effects of Environment

**DOI:** 10.3390/ijms252312571

**Published:** 2024-11-22

**Authors:** Yury Loika, Elena Loiko, Irina Culminskaya, Alexander M. Kulminski

**Affiliations:** Biodemography of Aging Research Unit, Social Science Research Institute, Duke University, Durham, NC 27705, USA; elena.loiko@duke.edu (E.L.); irina.kulminskaya@duke.edu (I.C.)

**Keywords:** genome-wide association study, Alzheimer’s disease, physical activity, exercises, pleiotropy, aging, sex, environment

## Abstract

Physical activity (PA) is a modifiable factor in mitigating/preventing Alzheimer’s disease (AD). It is crucial to identify the conditions under which PA’s effects on AD risk would be beneficial. This study aims to gain insights into pleiotropic predisposition to AD and PA within and across sexes and environmental effects. We performed a genome-wide association study (GWAS) of pleiotropic AD-PA associations in individuals (65 years and older) of European ancestry in a US sample (14,628 individuals), for men and women separately and combined, and contrasted them with the UK biobank (204,789 individuals) to elucidate the effects of the environment. Fisher’s method and Wald's test were used for estimating the significance of pleiotropic associations and differences between the samples. We identified genetic markers in 60 loci with significant pleiotropic associations. Of them, 91.7% of loci exhibited antagonistic relationships characterized by a misalignment of the signs of the associations of the same alleles with AD and PA and a correlation between these phenotypes. Only 16.7% of associations were replicated in the UKB. Phosphorylation and the regulation of transcription were identified as more pronounced biological mechanisms of AD-PA pleiotropy in females and males, respectively. Our results demonstrate the intrinsic heterogeneity of AD-PA pleiotropy and suggest that PA should be used as an intervention against AD with caution, after identifying groups of individuals and combinations of gene–environment interactions with beneficial effects.

## 1. Introduction

Physical activity (PA) improves cognitive function, memory, and learning, during all human life from childhood to old age [1,2]. PA is beneficial for healthy older adults, with modest improvements in executive function, motor function, cognitive speed, delayed memory functions, and auditory and visual attention [3,4]. PA and exercise can improve the cognition of older adults with Alzheimer’s disease (AD), dementia, and cognitive impairments as well [5,6]. Additionally, exercise generally reduces the risk of dementia and AD and the risk of cognitive decline [7,8,9].

There are many studies in humans and laboratory animals which attempt to investigate the potential mechanisms of the beneficial effects of exercise and to suggest the optimal PA intervention for older adults with AD and dementia [10,11,12]. How PA can prevent or delay the onset and progression of AD is not clear, but it is extremely important, because globally nearly 55 million people are now living with Alzheimer’s or related dementia and these numbers are rising [13].

The benefits of PA in cognition include increased cerebral blood flow, angiogenesis, neurogenesis, neurotransmission, synaptogenesis, and synaptic strength and plasticity; the imparting of substantial neuroprotection; enhanced levels of myokines, growth, and neurotrophic factors (Irisin, Cathepsin B, BDNF, IGF-1, LIF, L-Lactate, NGF, GDNF, VEGF); and decreased toxic Aβ and tau (key biomarkers of AD pathology) [14,15,16,17,18,19,20,21,22]. PA decreases free radicals’ production and oxidative stress and increases level of antioxidant enzymes (superoxide dismutase and catalase), and improves endothelial function by increasing levels of endothelial nitric oxide synthase (eNOS) [8,11]. PA improves the immune condition of the brain, and inhibits inflammation, microglia, and astrocyte activation [23,24]. In addition, PA improves glucose homeostasis by reducing insulin resistance, improving insulin sensitivity and increasing brain glucose uptake [25,26]. Also, PA and exercise reduce the risks of cardiovascular diseases, improve metabolic and lipid profiles, and reduce obesity and hypertension [27,28,29,30]. PA and diet can prevent or delay the onset of type 2 diabetes [31]. Since all the aforementioned risk factors are also risk factors for AD, managing them earlier in life may serve as a preventive intervention against AD. This underscores the importance of lifestyle modifications in reducing the risk of multiple conditions. Additionally, PA’s psychological benefits for mental health include reducing depression and anxiety, improving mood and sleep, and preventing other mental disorders [32,33,34].

It is evident that biological processes within the human body mediate the effects of PA/exercise on cognitive functions and memory, implying a genetic component that regulates these processes. Therefore, PA/exercise and pathological processes related to AD can affect the same network of genes involved in the biological pathways that regulate cognitive function and memory in humans. Identifying these genes and dissecting the complexity of their effects within this network can provide insights into how PA/exercise modulates AD risk. This understanding can highlight potential interventions involving PA and exercise to mitigate or even prevent the development and progression of AD.

The correlation between AD and PA may indicate common biological processes underlying both conditions. Studying patterns of the pleiotropic associations with AD and PA in different populations can facilitate identifying and understanding common and population-specific biological mechanisms underlying AD pathogenesis and the effects of PA on AD development and progression. Genome-wide association studies (GWASs) are comprehensive tools designed to identify genes and related biological mechanisms associated with different traits. In this paper, we use their pleiotropic extension to investigate genetic pleiotropy in the AD-PA pair of traits.

The goal of this study is to gain insights into pleiotropic predisposition to AD and PA, focusing on effects that are common for both sexes, sex-specific effects, and environmentally shaped effects. An additional goal was to investigate antagonistic genetic heterogeneity in this pair of traits. For this purpose, we carried out a pairwise pleiotropic genome-wide association study (~7 M common genetic variants) for predisposition to AD and PA in a sample of 14,628 individuals from three US-based cohorts and in a sample of ~204 K subjects from the UK Biobank. Fisher’s method was used as a test of the significance of pleiotropic associations and the Wald test was used to identify whether effects were significantly different between males and females and between US and UK samples.

## 2. Results

### 2.1. Study Overview

All analyses in this study were performed in three groups of European ancestry (Table 1): (1) males, (2) females, and (3) males and females combined (see Materials and Methods). Univariate GWAS was performed for each of the two traits, AD and PA, in each of the four cohorts separately. Then, a meta-analysis was performed to aggregate the results across three US cohorts for AD and PA separately and a pleiotropic test was performed by using Fisher’s method to combine *p*-values for AD and PA statistics. Additionally, the Wald test was applied in the US meta-samples to identify whether associations in males and females were significantly different.

**Figure 1:**
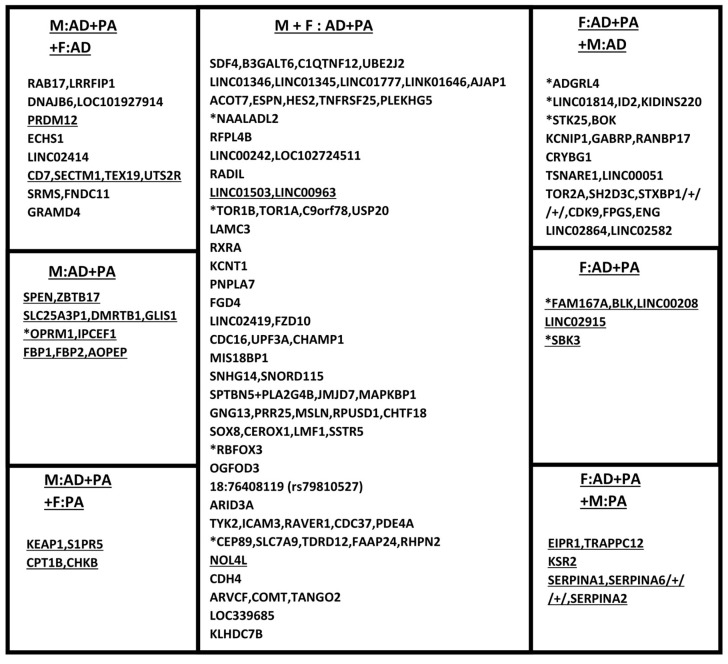
Genetic loci demonstrated significant pleiotropic associations with Alzheimer’s disease (AD) and physical activity (PA) in the US meta-analyzed cohorts. The central cell, M + F:AD + PA, includes loci in which SNPs demonstrated significant pleiotropic associations with AD and PA in the samples of males and females combined and there were no sex-specific univariate associations (see Methods). The middle-right and middle-left cells include loci with SNPs demonstrating significant pleiotropic associations with AD and PA in only one sex in females and males, respectively. Cells at the four corners include loci with SNPs demonstrating a significant sex-specific pleiotropic association in one sex and only one significant univariate association in the other sex. Specifically, they (upper-left corner, “M:AD + PA| +F:AD”) were pleiotropic in males and univariate with AD in females; (lower-left corner, “M:AD + PA| +F:PA”) pleiotropic in males and univariate with PA in females; (upper-right corner, “F:AD + PA| +M:AD”) pleiotropic in females and univariate with AD in males; and (lower-right corner, “F:AD + PA| +M:PA”) were pleiotropic in females and univariate with PA in males. Underlined loci indicate significantly different associations between males and females in the analysis of AD, PA, or both. An asterisk (*) denotes loci in which directions of the associations with AD and PA were replicated in the UKB sample. The symbol / + / indicates a continuation of the previous line.

Next, SNPs and respective genetic loci with significant pleiotropic associations in combined samples of males and females as well as SNPs with common effects in males and females (central cell in Figure 1) were identified. Then, we identified SNPs with significant sex-specific pleiotropic associations, which demonstrated significant associations with both traits in one sex group and with one trait in the other sex group (cells at four corners in Figure 1). Then, we identified SNPs demonstrating significant pleiotropic associations in one sex only, while in the other sex group both associations, with AD and PA, were non-significant (middle-left cell for males and middle-right cell for females in Figure 1).

In total, 60 genetic loci were identified (see Table S1 and Figure 1) in which SNPs demonstrated significant pleiotropic associations in the US meta-sample of males, females, and the two combined.

Finally, we compared effect directions of the SNP–trait associations from 60 genetic loci identified in the US sample with the same SNP–trait associations in respective UKB samples, and defined whether these pleiotropic associations were replicated.

### 2.2. Pleiotropic AD + PA Associations in the US Meta-Sample: Males and Females Combined

Significant pleiotropic associations with AD and PA (p_AD + PA_ ≤ 5 × 10^−6^, p_AD_ ≤ 5 × 10^−2^, and p_PA_ ≤ 5 × 10^−2^) were identified in 51 loci in the US meta-sample (see Section 4), for males and females combined (Table S1). In 32 of these loci, pleiotropic associations were sex-nonspecific. These loci are given in the central cell in Figure 1 (see Table S1 for details). Moreover, the associations of these SNPs with AD were significant at the level of *p* < 0.1 in males and females separately. The *NOL4L* gene was an exception from this rule. SNPs from this gene locus demonstrated significant association with AD in females, but with PA in males. By using the Wald test, a statistically significant difference between males and females in the association with AD was identified at two loci only (Table S1), *NOL4L* (p_Wald_ = 9.11 × 10^−3^) and *LINC01503*/*LINC00963* (p_Wald_ = 2.54 × 10^−2^). There was no significant difference in the associations with PA between males and females (Table S1).

### 2.3. Significant Pleiotropic Associations in One Sex and One Univariate Association in the Other Sex

We identified the genetic loci in which one of the four associations (i.e., two associations in each sex) was non-significant in the US meta-sample. This means that such SNPs demonstrated significant pleiotropic associations in one sex and a significant association with only one trait in the other sex. In Figure 1, they are presented in the upper- and lower-left cells for males and in the upper- and lower-right cells for females.

From 51 loci mentioned in the previous subsection, SNPs with this type of associations were observed in 19 loci. Nine and ten of them were specific to males and females, respectively. Additionally, we identified two genes with SNPs demonstrating significant pleiotropic associations in males (*CPT1B*/*CHKB*) and females (*KSR2*), but non-significant associations with AD in females and males, respectively. 

### 2.4. Sex-Specific Pleiotropic Associations in the US Meta-Sample

We identified genetic loci in which SNPs demonstrated significant pleiotropic associations with AD and PA in one sex only. Associations of the same SNPs with both AD and PA were non-significant in the other sex (see Table 2 and Table S1). In Figure 1, they are presented in the middle-left and middle-right cells for males and females, respectively. The Wald test (see Table 2 and Table S1) demonstrated at least a marginally significant difference between males and females in the associations of all these SNPs with AD and for four SNPs (mapped to *SLC25A3P1*/*DMRTB1*/*GLIS1*, *OPRM1*/*IPCEF1*, *FAM167A*/*BLK*/*LINC00208*, and *SBK3* gene clusters) with PA. All significant pleiotropic associations of these SNPs were antagonistic, meaning the sign of the product of the effects for associations with AD and PA was opposite to the sign of the correlation coefficient between these traits.

### 2.5. Abundant Antagonistic Pleiotropic Associations with AD and PA

Our analysis identified that SNPs in 55 loci [91.7% of all identified loci] demonstrated antagonistic pleiotropic associations (see Table S1). Non-antagonistic pleiotropic associations with AD and PA were demonstrated in five genetic loci only (see Table 3 and Table S1, and Figure 1). The latter were observed in both sexes in two loci (*RFPL4B* and *PGD4*), in males in one locus (*KEAP1*/*S1PR5*), and in females in two loci (*CRYBG1* and *KSR2*). Neither of these non-antagonistic associations were replicated in the UKB sample (Table S2).

### 2.6. Replication of Pleiotropic Associations in UKB

We considered the replication of pleiotropic associations that attained the required level of significance in the US meta-sample (see Materials and Methods). For common associations across sexes (central cell in Figure 1), the replication of the directions of associations with AD and PA was tested in the UKB sample of males and females combined. For sex-specific pleiotropic associations—left (right) column of cells for males (females) in Figure 1—we tested the replication in the corresponding UKB sex sample.

The results of our replication analysis are presented in Table 4 (see also Table S2). Significant pleiotropic associations identified in the US meta-samples were replicated in the UKB samples for SNPs in 10 loci only [16.7% of all loci]. Our analysis identified the replication of pleiotropic associations in four loci (*NAALADL2*, *TOR1B*/*TOR1A*/*Corf78*/*USP20*, *RBFOX3*, and *CEP89*/*SLC7A9*/*TDRD12*/*FAAP24*/*RHPN2*) in the combined sample of males and females (central cell in Figure 1), in five loci (*ADGRL4*, *LINC01814*/*ID2*/*KIDINS220*, *STK25*/*BOK*, *FAM167A*/*BLK*/*LINC00208*, and *SBK3*) in females (cells on the right in Figure 1), and in one locus (*OPRM1*/*IPCEF1*) in males (cells on the left in Figure 1).

Replicated association for an SNP mapped to the *NAALDL2* gene demonstrated significant pleiotropic associations in the UKB sample of males and females combined (Table 4 and Table S2).

## 3. Discussion

We performed pairwise pleiotropic analysis of the associations of SNPs with AD and PA in the US meta-samples of males and females, separately and combined, which comprised three cohorts (ARIC, CHS, and FHS). Our analysis identified 60 genetic loci in which SNPs demonstrated significant pleiotropic associations that were common in both sexes or specific to one sex with no or one association (either with AD or PA) in the other sex. These SNPs were associated with AD and PA at *p* < 5 × 10^−2^ and attained *p* < 5 × 10^−6^ in pleiotropic analysis. Also, we tested the replication of the identified associations in the UKB cohort. Of 60 loci, 32 loci were common for both sexes. Seven genetic loci demonstrated significant pleiotropic associations in one sex only. Also, we identified eight (eight) and two (three) genetic loci with sex-specific significant pleiotropic associations in males (females), which demonstrated significant associations in females (males) with one trait only, AD and PA, respectively. All these loci are depicted in four corners in Figure 1. In total, 28 sex-specific loci were identified with significant pleiotropic associations: 14 in males (three left cells in Figure 1) and 14 in females (three right cells in Figure 1).

### 3.1. Sex-Specific Pleiotropic Associations with AD and PA

Of seven genetic loci with significant pleiotropic associations in one sex only, four loci were identified in males (*SPEN*/*ZBTB17*; *SLC25A3P1*/*DMRTB1*/*GLIS1*; *OPRM1*/*IPCEF1*; *FBP1*/*FBP2*/*AOPEP*) and three in females (*FAM167A*/*BLK*/*LINC00208*; *LINC02915*; and *SBK3*). 

### 3.2. Phosphorylation as Female-Specific Process in AD-PA Pleiotropy 

Genes at two of the three loci with significant pleiotropic associations specific to females were involved in phosphorylation (*SBK3*, *BLK*). *SBK3* (SH3 domain binding kinase family member 3) is a member of the protein serine/threonine kinase family (see middle-right cell in Figure 1). The *SBK3* gene’s highest level of expression is in human cardiac muscle tissue. *BLK* (BLK Proto-Oncogene, Src Family Tyrosine Kinase), nonreceptor tyrosine-kinase, enhances insulin synthesis and secretion in response to glucose in beta-cells [35]. Both genes encode protein kinases involved in protein phosphorylation, a post-translational modification that regulates many cellular functions, such as cell differentiation, proliferation, growth, apoptosis, and signaling [36]. 

When other genes with significant pleiotropic associations in females (three right cells in Figure 1) were considered, the number of genes related to the phosphorylation processes is further extended by genes in three loci: *SH2D3C*/*CDK9*/*ENG*, *STK25*, and *KSR2*. *CDK9*, serine/threonine protein kinase, is involved in DNA repair. *SH2D3C* (SH2 domain containing 3C) and *ENG* (Endoglin) are associated with phosphorylation too: *SH2D3C* is involved in the positive regulation of peptidyl-serine phosphorylation. *ENG* encodes a protein that is itself a target for phosphorylation and plays a role in angiogenesis. *STK25* is a serine/threonine protein kinase involved in the response to environmental stress and axonogenesis. *KSR2* (kinase suppressor of Ras 2) is a scaffold protein, bringing together the key components of the Raf/MEK/ERK (MAPK) pathway. In this pathway, extracellular-regulated kinase (ERK2), an MAP kinase, can phosphorylate tau [37]. 

In the set of 14 genetic loci with sex-specific significant pleiotropic associations in males (three cells on the left side in Figure 1), we identified only two genes/loci related to phosphorylation, namely *SRMS* (Src-related kinase lacking C-terminal regulatory tyrosine) and *CHKB* (choline kinase beta). The *SRMS* gene is involved in peptidyl-tyrosine autophosphorylation. Choline kinase (CK) and ethanolamine kinase (EK) catalyze the phosphorylation of choline/ethanolamine to phosphocholine/phosphoethanolamine.

Phosphorylation is one of the key processes involved in AD pathogenesis. The abnormal phosphorylation of tau proteins is a hallmark of AD and its accumulation leads to the development of different neuropathological conditions [38]. Protein phosphorylation plays a key role in the response of the human body to stress associated with exercise and physical activity and is related to the MAPK and NF-κB signaling pathways [39]. Therefore, phosphorylation processes can be a link in pleiotropic associations with AD and PA. Our analysis identified five and two genetic loci with sex-specific pleiotropic associations in females and males, respectively, which are related to phosphorylation processes. Therefore, our results suggest that the AD-PA pleiotropic effects related to phosphorylation are more pronounced in females than in males. Moreover, the replication of pleiotropic associations in the UKB cohort for two of the female-specific genes related to phosphorylation (*BLK* and *SBK3*, see middle-right cell in Figure 1) suggests sex-specific effects of phosphorylation in females across a spectrum of exogenous exposures, as shown in the US vs. UK case considered in this paper.

### 3.3. Transcription as Male-Specific Process in AD-PA Pleiotropy

Genes at three of the four loci with significant pleiotropic associations specific to males (middle-left cell in Figure 1) were involved in the regulation of transcription (DNA-templated transcription and transcription by RNA Polymerase II). This set includes *SPEN*, *ZBTB17*, *GLIS1*, *DMRTB1*, and *FBP1*. *SPEN*, *ZBTB17*, *GLIS1*, and *DMRTB1* encode transcription factors that can play both roles, as a repressor and an activator of transcription, which is the first step in gene expression. Associations between SNPs in *GLIS1*, *DMRTB1*, and amyloid beta were recently reported in GWAS [40]. 

*SPEN* suppresses the transactivation activity of Notch signaling. Altered Notch signaling may be associated with the development of AD through its role in cerebral vasculature [41]. *SPEN* can affect adult neurogenesis because Notch signaling is involved in neuronal arborization, the integration of new neurons, and stem-cell quiescence [42,43]. It was shown that adult hippocampal neurogenesis was reduced in patients with AD and the neural stem cells were in a more quiescent state [44]. *OPRM1* encodes the mu opioid receptor, which is the primary receptor for endogenous and exogenous opioids and is responsible for pain relief. The increased methylation of the *OPRM1* gene in AD patients was shown in [45]. *FBP1* and *FBP2*, Fructose-Bisphosphatase 1 and 2, encode regulatory enzymes of glycolysis, gluconeogenesis, carbohydrate, and fructose metabolism. Glycolytic dysfunction may cause the development of AD pathologies [46].

In the set of 14 genetic loci with sex-specific significant pleiotropic associations in females (three cells on the right side in Figure 1), we identified only one gene/locus related to transcription, *ID2* (inhibitor of DNA binding 2), a transcription factor, regulating a variety of cellular processes, including cellular growth, senescence, differentiation, apoptosis, angiogenesis, and neoplastic transformation.

Therefore, our results suggest that the AD-PA pleiotropic effects related to the regulation of transcription are more pronounced in males than in females. Sex-specific pleiotropic associations in males identified in the US meta-samples (middle-left cell in Figure 1) were replicated in the UKB cohort in one locus only (*OPRM1*/*IPCEF1*). This suggests that regulation of transcription in biological mechanisms related to AD-PA pleiotropy can be subjected to pronounced effects of exogenous exposures. 

### 3.4. Antagonistic Genetic Heterogeneity Was Observed for Pleiotropic Associations with AD and PA 

Antagonistic genetic heterogeneity is characterized by a misalignment of the signs of the product of the effects of genetic associations with different phenotypes and the correlation between these phenotypes [47,48,49,50,51]. In all US cohorts—males and females, both separately and combined (except males in the CHS)—negative correlation was observed between AD and PA. In this context, antagonistic heterogeneity means that SNPs with such a property should demonstrate the same directions of their associations with AD and PA in the US samples. The same allele of such SNPs should be associated with increased (decreased) PA and an increased (decreased) risk of AD. In this study, SNPs in 55 of 60 identified loci, i.e., 91.7% of the identified SNPs with significant pleiotropic effects, demonstrated genetic heterogeneity. These findings indicate that biological mechanisms (related to the identified genes) that support higher levels of PA can also be associated with increased AD risks. This result does not support the previous reports of unconditional benefits of PA in improving cognition and preventing AD.

### 3.5. Replication and Heterogeneity of Pleiotropic Associations Related to Exogenous Exposures

Only 10 out of 60 (16.7%) significant pleiotropic associations identified in the US samples were replicated in the respective UKB samples. All 10 replicated associations demonstrated genetic heterogeneity, which suggests a replication of the adverse effects of high levels of PA on AD risks. A small percentage of replicated significant pleiotropic associations indicate that biological mechanisms linking PA and the risk of AD are shaped by exogenous exposures. Some of the results in Table S2 support this conclusion. For instance, in males, the directions of the associations with PA of the same alleles of rs10245704 (*DNAJB6*/*LOC101927914* locus on chr.7) and rs1999224 (*TOR2A*/*SH2D3C*/*STXBP1*-/*CDK9*/*FPGS*/*ENG* gene cluster on chr.9) were replicated in the UK cohort, while associations of the same alleles with AD were of opposite directions in the UK and US meta-samples. Moreover, the associations of these SNPs with AD were significant in samples from both countries. In particular, the minor allele of rs10245704 (*DNAJB6*/*LOC101927914*) demonstrated at least a marginally significant negative association with PA in both UK (β_PA_ = −0.034, p_PA_ = 8.72 × 10^−2^) and US (β_PA_ = −0.167, p_PA_ = 8.46 × 10^−3^) samples and were associated with an increased risk of AD in the UKB (β_AD_ = 0.153, p_AD_ = 8.16 × 10^−2^) and a decreased risk of AD in the US meta-sample (β_AD_ = −0.367, p_AD_ = 3.27 × 10^−2^). This result indicates that the same mechanisms that lead to, or are involved in, higher PA can demonstrate adverse or protective effects on AD risk. This difference can likely be shaped by exogenous exposures, which, in our case, are attributed to different countries, the UK and US.

## 4. Materials and Methods

### 4.1. Accession Numbers

This manuscript was prepared using limited-access data obtained though dbGaP from the ARIC (phs000280.v7.p1), CHS (phs000287.v7.p1), and FHS (phs000007.v32.p13). Also, this research was conducted using data from UK Biobank, a major biomedical database (http://www.ukbiobank.ac.uk/), accessed on 15 March 2021.

### 4.2. Study Cohorts

In this paper, we analyzed data on individuals of Caucasian ancestry who were 65 years and older, drawn from four longitudinal studies. One sample was from the UK Biobank (UKB) [52,53], and the other three represented the US meta-sample, comprising the Atherosclerosis Risk in Communities (ARIC) study [54], Cardiovascular Health Study (CHS) [55], and Framingham Heart Study (FHS) parental and offspring cohorts [56]. Table 1 presents basic demographic information for the genotyped participants in these studies. 

### 4.3. Genotypes

To perform the meta-analysis in the US sample and to compare results between the US and UK samples, we used imputed genetic data after harmonization. The imputed genetic data for the UKB participants were provided by the UKB team and were obtained by using the Haplotype Reference Consortium (HRC) and UK10K imputation panels. For participants from the US cohorts, the imputation process was performed at the Michigan Imputation Server (MIS) by using the HRC reference panel and data from the following genotyping arrays: Affymetrix 6.0 chip (~1 M SNPs) in the ARIC study, Illumina HumanCNV370v1 chip (~370 K SNPs) in the CHS, and Affymetrix 500 K chip (~500 K SNPs) in the FHS cohorts. Rayner’s tools with the HRC reference panel (https://www.well.ox.ac.uk/~wrayner/tools/) were used for the quality control of genetic data submitted to the MIS. In the analyses, imputed genetic markers with a minor allele frequency (MAF) greater than 0.5% were included, which resulted in ~7 M overlapping genetic variants. 

### 4.4. Phenotypes

We used information about AD affection status provided by the investigators, which was based on the International Classification of Disease (ICD) tenth revision (ICD-10), G30 and F00 codes in the UKB, the ICD ninth revision ICD-9, the 331.0 code in CHS, and both ICD-9 and ICD-10 codes and death certificates in the ARIC. In the FHS, AD affection status was defined by using the National Institute of Neurological and Communicative Disorders and Stroke and the Alzheimer’s Disease and Related Disorders Association criteria [57].

Dichotomized variables were used for physical activity, identifying low and high PA levels. In the UKB, the metabolic equivalent of task (MET)-minutes per week measure was used to identify participants with a low (MET < 1500) and high (MET ≥ 1500) level of PA. 

In the FHS, PA level was defined based on a PA index (PAI) introduced and provided by the FHS investigators [58,59]. We used information for PAI measured at exam 12 and exam 7 for the parental (age group of 40–88 years old) and offspring (age group of 50–83 years old) cohorts, respectively. A high (low) PA level was defined as PAI ≥ 33 (PAI < 33).

In the ARIC study, the investigators estimated PA by using a questionnaire [60]. A score of PA at work (“work score”) was used in the ARIC study because it was the main type of PA in the selected age group of participants (45–64 years old). A high (low) PA level was determined as a “work score” ≥ (<) 2.3 at the baseline.

Since the CHS included individuals older than 65 years, the CHS investigators used information about 15 leisure activities at different examinations to calculate MET-minutes per week and to identify individuals with low, moderate, and heavy leisure activity levels. CHS participants with a high (low) PA level were determined as those with heavy (low or moderate) leisure activity levels at the baseline (the first exam).

### 4.5. Correlation Between AD and PA

Information on Pearson correlation coefficients between AD and PA is given in Table 1. A small positive correlation between AD and PA was observed in the UKB cohort. The largest magnitude of correlation was observed in the FHS cohort, with a negative direction in males, females, and the combined sample. In the CHS cohort, the correlation was positive in males but negative in females. In the ARIC cohort, the correlation coefficient was moderate in magnitude and negative in direction in males, females, and the combined sample.

### 4.6. Statistical Analyses

***The univariate unconditional GWAS*** was performed in six samples in each study, i.e., for AD and PA separately in samples of males, females, and males and females combined. We used a logistic regression model, as implemented in *plink 2.0* software (version alpha 6, 20231212) and considered an additive genetic model for the minor allele as an effect allele. All models were adjusted for sex (in combined samples) and age at the selected examination, as well as for cohort (parents and offsprings) in the FHS dataset. 

***Univariate meta-analysis*** was performed for each SNP–trait association by pooling results from three US cohorts using fixed-effect meta-regression with inverse-variance weighting, as implemented in METAL software (version: generic-metal-2011-03-25.tar.gz) [61] (https://csg.sph.umich.edu/abecasis/Metal/ accessed on 23 February 2020), for each sex separately and in the combined sample. Wald’s test was used to estimate whether the effects were significantly different between the samples.

***Pleiotropic meta-analysis*** was performed for each SNP in the UKB and US meta-sample separately, to estimate the significance of SNP associations with AD and PA. Fisher’s method [62] was used for this analysis, as justified by the small correlation between AD and PA (Table 1). Fisher’s method combines *p*-values across phenotypes, disregarding the effect directions and correlation between them. Fisher’s method corrects pleiotropic *p*-values for testing multiple phenotypes.

### 4.7. Pleiotropic Associations

To characterize pleiotropic associations, we required the significance to be at *p* < *p_PL_* = 5 × 10^−6^ in the pleiotropic meta-analysis and *p* < *p_UV_* = 5 × 10^−2^ in the univariate analysis of AD and PA in males, females, or the combined group in the US meta-sample. Additionally, if an SNP demonstrated less significant univariate association in the combined sample of males and females than in males and females separately, this SNP was considered as specific to the sex, which was characterized by the most significant univariate association, unless explicitly stated. Otherwise, it was considered as sex-nonspecific. 

### 4.8. Replication of Pleiotropic Associations

Additionally, we report the replication of the associations of significant pleiotropic SNPs discovered in the US samples within the UKB cohort, analyzing males and females separately and combined. We assumed that a pleiotropic association of an SNP was replicated in the UKB if the associations of this SNP with AD and PA in the corresponding UKB sample (males and females, separately or combined) demonstrated the same effect directions as in the US sample, disregarding the significance of these associations in the UKB samples. To avoid uncertainty due to negligible effects, we considered the associations with promising effects for a value of |β| ≥ 0.01 only.

### 4.9. Index SNPs and Gene Mapping

The NCBI dbSNP database (https://www.ncbi.nlm.nih.gov/snp) and variant effect predictor from Ensembl (assembly GRCh37.p13, https://grch37.ensembl.org/Homo_sapiens/Info/Index) were used for mapping SNPs to genes. One index SNP was selected per locus. For SNPs that were not mapped to a gene, the closest genes were reported. If an index SNP was in linkage disequilibrium (r^2^ > 0.8) with the other SNPs in other genes within a ±500 Kbp region, we reported these genes as well. For this purpose, we used LD link tools [63] from NIH (http://ldlink.nih.gov).

## 5. Conclusions

This study highlights the complex contribution of genetic factors in the link between PA and the risks of AD.

We identified sex-specific genetic links between AD and PA. Our results suggest that the AD-PA pleiotropic effects related to the regulation of transcription are more pronounced in males than in females, while phosphorylation is more pronounced in females than in males. The latter indicates that one additional layer in the regulation of gene expression, namely, feedback on transcription through phosphorylation, is more pronounced in females than in males and can help regulate the link between PA and AD. 

In total, 91.7% of the identified significant pleiotropic associations in the US samples demonstrated antagonistic genetic heterogeneity, which means that the same alleles were associated with high levels of PA and increased risks of AD. This finding does not support the hypothesis about the unconditional protective effect of PA on AD.

Only 16.7% of pleiotropic associations with AD and PA that were significant in the US samples were replicated in the respective samples from the UKB. Some of the genetic markers demonstrated opposite directions in their associations with AD in the UKB, as compared to the respective US samples, despite showing the same directions of their associations with PA in both countries. This indicates that the effects of PA on the risks of AD are likely shaped by exogenous exposures. 

Therefore, the results reported here suggest that cautions should be taken when using PA and exercises as a preventive tool for decreasing the risks of AD. This tool should be tailored to particular populations under certain types of exogenous exposures and other conditions. Further research is required to identify groups of individuals with more homogeneous genetic effects as well as all related conditions and peculiarities that will be beneficial or can cause any adverse effects related to AD.

This study reported results on sex-specific and common pleiotropic AD + PA associations in individuals of European ancestry, the largest race/ethnic group in the investigated datasets. Considering the differences and peculiarities of metabolic processes in different race/ethnic groups, the reported results cannot be unconditionally translated to other race/ethnic groups. Further research is required to identify and highlight respective similarities and differences.

## 6. Limitations

We acknowledge the study limitations. First, it considered samples of European ancestry only, which limits the translation of the results to other race/ethnic groups. Second, this study did not stratify by age, which could highlight age-specific genetic associations and respective biological mechanisms.

## Figures and Tables

**Table 1:** Basic demographic information for the UK and US participants with available genotyping information.

PAR	UKB	US	ARIC	CHS	FHS
*n*	204,789	14,628	7319	3277	4032
Women (%)	103,532 (50.6)	8077 (55.2)	3838 (52.4)	1973 (60.2)	2266 (56.2)
BCrange	1934–1956	1885–1950	1922–1944	1890–1925	1885–1950
Age at exam	62.71	60.57	54.58	72.41	61.83
Age at follow-up	71.50	81.10	79.88	83.62	81.26
Mortality (%)	21,108 (10.3)	6857 (46.9)	3234 (44.2)	1457 (44.5)	2166 (53.7)
AD cases (%)	2096 (1.0)	977 (6.7)	281 (3.8)	204 (6.2)	492 (12.2)
High PA (%)	82,429 (40.3)	6987 (47.8)	3098 (42.3)	1502 (45.8)	2387 (59.2)
Ever Smoke (%)	102,322 (50.0)	8626 (59.0)	4382 (59.9)	1716 (52.4)	2528 (62.7)
Correlation coefficient AD|PA, [%]					
M + F [%]	0.29		−3.01	−0.40	−22
M [%]	0.20		−3.48	1.47	−6.53
F [%]	0.35		−2.09	−1.29	−14.46

PAR = name of characteristic/parameter; N = sample size; women = number of women (%); BCrange = range of birth years; age at exam = mean age measured in years at selected examination of PA; age at follow-up = life span in years at end of follow up or death. AD = Alzheimer’s Disease; PA = Physical Activity. M + F = combined sample of males and females; M = males; F = females.

**Table 2:** SNPs demonstrated sex-specific significant pleiotropic associations with AD and PA in the US meta-sample.

CHR:POS. rsID_EE	Gene Cluster	Sex	P_F_	AD				PA			
				Beta	SE	*p*	P_W_	Beta	SE	*p*	P_W_
1:16240949 rs9633365_t	*SPEN* *ZBTB17*	F	2.03 × 10^−1^	−0.091	0.069	1.91 × 10^−1^	2.50 × 10^−6^	0.040	0.036	2.68 × 10^−1^	3.00 × 10^−2^
		M	1.36 × 10^−7^	0.421	0.084	4.90 × 10^−7^		0.095	0.039	1.40 × 10^−2^	
		F + M	3.63 × 10^−3^	0.110	0.053	3.83 × 10^−2^		0.067	0.026	1.08 × 10^−2^	
1: 53955202 rs1288615_c	*SLC25A3P1* *DMRTB1* *GLIS1*	F	7.77 × 10^−1^	−0.019	0.083	8.24 × 10^−1^	1.31 × 10^−2^	0.031	0.047	5.00 × 10^−1^	4.98 × 10^−4^
		M	9.99 × 10^−7^	−0.381	0.120	1.54 × 10^−3^		−0.207	0.050	3.66 × 10^−5^	
		F + M	6.08 × 10^−3^	−0.146	0.068	3.09 × 10^−2^		−0.077	0.034	2.40 × 10^−2^	
6: 154471195 rs13196610_g	*OPRM1* *IPCEF1*	F	8.25 × 10^−1^	−0.026	0.131	8.41 × 10^−1^	2.99 × 10^−3^	0.039	0.067	5.59 × 10^−1^	8.30 × 10^−2^
		M	4.58 × 10^−6^	0.538	0.137	9.00 × 10^−5^		0.209	0.071	3.16 × 10^−3^	
		F + M	3.17 × 10^−3^	0.214	0.095	2.41 × 10^−2^		0.118	0.049	1.47 × 10^−2^	
9: 97418573 rs41281162_a	*FBP1* *FBP2* *AOPEP*	F	1.54 × 10^−1^	−0.279	0.198	1.59 × 10^−1^	3.48 × 10^−5^	0.099	0.081	2.23 × 10^−1^	5.23 × 10^−1^
		M	1.72 × 10^−6^	0.802	0.170	2.32 × 10^−6^		0.175	0.087	4.33 × 10^−2^	
		F + M	1.05 × 10^−2^	−0.279	0.198	1.59 × 10^−1^		0.135	0.059	2.27 × 10^−2^	
8: 11332026 rs12680762_t	*FAM167A* *BLK* *LINC00208*	F	2.19 × 10^−6^	−0.170	0.070	1.57 × 10^−2^	7.16 × 10^−2^	−0.165	0.037	8.29 × 10^−6^	1.08 × 10^−2^
		M	7.14 × 10^−1^	0.036	0.090	6.93 × 10^−1^		−0.027	0.040	5.00 × 10^−1^	
		F + M	2.49 × 10^−4^	−0.094	0.055	9.10 × 10^−2^		−0.099	0.027	2.33 × 10^−4^	
15: 39595347 rs62002283_g	*LINC02915*	F	1.38 × 10^−6^	−0.417	0.106	8.15 × 10^−5^	7.79 × 10^−3^	−0.166	0.050	9.79 × 10^−4^	1.67 × 10^−1^
		M	5.60 × 10^−1^	0.017	0.124	8.93 × 10^−1^		−0.063	0.055	2.52 × 10^−1^	
		F + M	3.90 × 10^−5^	−0.250	0.080	1.83 × 10^−3^		−0.117	0.037	1.55 × 10^−3^	
19: 56056676 rs595284_a	*SBK3*	F	9.86 × 10^−7^	0.443	0.101	1.25 × 10^−5^	7.19 × 10^−2^	0.166	0.059	4.47 × 10^−3^	7.23 × 10^−2^
		M	7.17 × 10^−1^	0.126	0.144	3.84 × 10^−1^		0.007	0.066	9.10 × 10^−1^	
		F + M	3.40 × 10^−5^	0.327	0.083	8.35 × 10^−5^		0.095	0.044	2.93 × 10^−2^	

CHR:POS = chromosome:position (GRCh37) of considered SNP; rsID_EA = rs identifier and the effect allele of considered SNP; gene cluster = mapped or closest genes to identified SNP; sex = sex of considered sample [M = males, F = females, M + F = males and females combined]; P_F_ = *p*-value for pleiotropic association with AD and PA obtained by Fisher’s method; beta = effect size; SE = standard error; *p* = *p*-value; P_W_ = *p*-value from the Wald test for significant difference in effects in males vs females. AD = Alzheimer’s disease; PA = physical activity. Gray color highlights sex-specific significant pleiotropic associations.

**Table 3:** SNPs demonstrated non-antagonistic pleiotropic associations with AD and PA in the US meta sample.

CHR:POS rsID_AE	Gene Cluster	Sex	P_F_	AD			PA		
				Beta	SE	*p*	Beta	SE	*p*
6:112347003 rs11153330_g	*RFPL4B*	F + M	1.87 × 10^−7^	0.264	0.051	2.55 × 10^−7^	−0.054	0.026	3.76 × 10^−2^
12:32640591 rs112595860_g	*FGD4*	F + M	2.46 × 10^−6^	0.265	0.058	3.96 × 10^−6^	−0.062	0.030	3.72 × 10^−2^
6:106974933 rs9486379_g	*CRYBG1*	F	8.07 × 10^−6^	0.158	0.069	2.29 × 10^−2^	−0.164	0.039	2.28 × 10^−5^
12:118129940 rs73213813_c	*KSR2*	F	4.08 × 10^−6^	0.340	0.086	7.86 × 10^−5^	−0.145	0.049	3.21 × 10^−3^
19:10616403 rs62131895_c	*KEAP1* *S1PR5*	M	3.33 × 10^−6^	0.456	0.100	5.18 × 10^−6^	−0.098	0.047	3.92 × 10^−2^

CHR:POS = chromosome:position (GRCh37) of considered SNP; rsID_EA = rs identifier and the effect allele of considered SNP; gene cluster = mapped or closest genes to identified SNP; sex = sex of considered sample [M = males, F = females, M + F = males and females combined]; P_F_ = *p*-value for pleiotropic association with AD and PA obtained by Fisher’s method; beta = effect size; SE = standard error; *p* = *p*-value. AD = Alzheimer’s disease; PA = physical activity.

**Table 4:** Replication of significant pleiotropic associations with AD and PA in UKB sample.

CHR:POS rsID_AE	Gene Cluster	Sex	Sample	P_F_	AD			PA		
					Beta	SE	*p*	Beta	SE	*p*
1: 79432703 rs12120090_t	*ADGRL4*	F	US	1.27 × 10^−5^	−0.491	0.128	1.17 × 10^−4^	−0.191	0.071	7.27 × 10^−3^
			UK	7.01 × 10^−1^	−0.062	0.102	5.45 × 10^−1^	−0.010	0.020	6.15 × 10^−1^
2:8728459 rs11680715_c	*LINC01814*, *ID2*, *KIDINS220*	F	US	2.04 × 10^−5^	−0.296	0.072	3.59 × 10^−5^	−0.085	0.041	3.94 × 10^−2^
			UK	7.72 × 10^−1^	−0.031	0.060	6.11 × 10^−1^	−0.005	0.012	6.65 × 10^−1^
2:242465295 rs11884861_g	*STK25*, *BOK*	F	US	4.39 × 10^−5^	−0.243	0.068	3.40 × 10^−4^	−0.097	0.037	9.46 × 10^−3^
			UK	6.32 × 10^−1^	−0.055	0.053	2.94 × 10^−1^	−0.001	0.010	9.43 × 10^−1^
3:175301950 rs6803414_c	*NAALADL2*	M + F	US	3.01 × 10^−6^	−0.301	0.065	4.06 × 10^−6^	−0.062	0.031	4.50 × 10^−2^
			UK	4.56 × 10^−2^	−0.057	0.041	1.63 × 10^−1^	−0.016	0.008	4.79 × 10^−2^
6:154471195 rs13196610_g	*OPRM1*, *IPCEF1*	M	US	4.58 × 10^−6^	0.538	0.137	9.00 × 10^−5^	0.209	0.071	3.16 × 10^−3^
			UK	8.85 × 10^−1^	0.033	0.087	7.02 × 10^−1^	0.005	0.018	7.98 × 10^−1^
8:11332026 rs12680762_t	*FAM167A*, *BLK*, *LINC00208*	F	US	2.19 × 10^−6^	−0.170	0.070	1.57 × 10^−2^	−0.165	0.037	8.29 × 10^−6^
			UK	4.17 × 10^−1^	−0.059	0.051	2.47 × 10^−1^	−0.006	0.010	5.70 × 10^−1^
9:132549841 rs78011773_a	*TOR1B*, *TOR1A*, *C9orf78*, *USP20*	M + F	US	5.38 × 10^−7^	−0.476	0.104	4.84 × 10^−6^	−0.139	0.050	6.06 × 10^−3^
			UK	4.52 × 10^−1^	−0.018	0.065	7.85 × 10^−1^	−0.017	0.013	2.03 × 10^−1^
17:77136678 rs1795952_c	*RBFOX3*	M + F	US	7.31 × 10^−7^	0.401	0.096	3.06 × 10^−5^	0.171	0.053	1.33 × 10^−3^
			UK	4.38 × 10^−1^	0.045	0.070	5.21 × 10^−1^	0.015	0.014	2.91 × 10^−1^
19:33364389 rs74646163_c	*CEP89*, *SLC7A9*, *TDRD12*, *FAAP24*, *RHPN2*	M + F	US	9.75 × 10^−7^	0.382	0.096	7.18 × 10^−5^	0.171	0.051	7.67 × 10^−4^
			UK	1.23 × 10^−1^	0.076	0.060	2.05 × 10^−1^	0.019	0.013	1.30 × 10^−1^
19:56056676 rs595284_a	*SBK3*	F	US	9.86 × 10^−7^	0.443	0.101	1.25 × 10^−5^	0.166	0.059	4.47 × 10^−3^
			UK	6.11 × 10^−1^	0.036	0.089	6.89 × 10^−1^	0.016	0.018	3.78 × 10^−1^

CHR:POS = chromosome:position (GRCh37) of considered SNP; rsID_EA = rs identifier and the effect allele of considered SNP; gene cluster = mapped or closest genes to identified SNP; sex = sex of considered sample [M = males, F = females, M + F = males and females combined]; P_F_ = *p*-value for pleiotropic association with AD and PA obtained by Fisher’s method; beta = effect size; SE = standard error; *p* = *p*-value. AD = Alzheimer’s disease; PA = physical activity.

## Data Availability

This article employs a secondary analysis of data obtained from dbGaP and the UK Biobank. The results are available in the main text of this article and in the Supplementary Materials. This manuscript was prepared using limited-access data obtained though dbGaP (accession numbers: phs000280.v7 [ARIC], phs000287.v7 [CHS], and phs000007.v32 [FHS]. This manuscript was prepared using limited-access phenotypic and genetic datasets of the UK Biobank, which are available through the following link: http://www.ukbiobank.ac.uk/.

## Supplementary Materials

The following supporting information can be downloaded at https://www.mdpi.com/article/10.3390/ijms252312571/s1.
