# Supplementary material for: Pleiotropic Associations with Alzheimer’s Disease and Physical Activity: Sex Differences and the Effects of Environment
Source: Int J Mol Sci. 2024 Nov 22;25(23):12571. doi: 10.3390/ijms252312571 (PMC11641332; doi:10.3390/ijms252312571)
Supplement: Supplementary file 1 [file ijms-25-12571-s001.zip › ijms-3262833-supplementary/ijms-3262833-supplementary/Supplementary Section S1.pdf]

Pleiotropic Associations with Alzheimer's Disease and Physical Activity: Sex Differences and the Effects of Environment

Yury Loika[1*], Elena Loiko[1], Irina Culminskaya[1], and Alexander M Kulminski[1*]

[1]Biodemography of Aging Research Unit, Social Science Research Institute, Duke University, Durham, NC, USA.

* Corresponding author(s).

SUPPLEMENTARY INFORMATION

Supplementary information includes one Text file and two Tables:

1. Supplementary Text

Text S1: Supplementary Acknowledgment Text

2. Supplementary Tables

Table S1: Univariate and pair-wise pleiotropic associations of selected SNPs with AD and PA in the US cohorts, males and females separately and combined.

Table S2: Pair-wise pleiotropic associations of selected SNPs with AD and PA and their comparison in the UKB and US meta sample, males and females separately and combined.

1. Supplementary Text

Text S1: Supplementary Acknowledgment Text.

This work was supported by the National Institute on Aging (NIA) (grant numbers R01AG065477, and R01AG070488), NIH, USA. The funding source did not participate in the writing of the manuscript or the decision to submit the manuscript for publication. The content is solely the responsibility of the authors and does not necessarily represent the official views of the National Institutes of Health (NIH). This manuscript was prepared using limited access data obtained though dbGaP (accession numbers: phs000280.v7 [ARIC], phs000287.v7 [CHS], and phs000007.v32 [FHS]. This manuscript was prepared using limited access phenotypic and genetic datasets of the UK Biobank, which are available through the following link: http://www.ukbiobank.ac.uk/

The Atherosclerosis Risk in Communities Study (ARIC) is carried out as a collaborative study supported by the NHLBI contracts (HHSN268201100005C, HHSN268201100006C, HHSN268201100007C, HHSN268201100008C, HHSN268201100009C, HHSN268201100010C, HHSN268201100011C, and HHSN268201100012C). The authors thank the staff and participants of the ARIC study for their important contributions. Funding for CARe genotyping was provided by NHLBI Contract N01-HC-65226.

The Cardiovascular Health Study (CHS) was supported by contracts HHSN268201200036C, HHSN268200800007C, N01-HC-85079, N01-HC-85080, N01-HC-85081, N01-HC-85082, N01-HC-85083, N01-HC-85084, N01-HC-85085, N01-HC-85086, N01-HC-35129, N01 HC-15103, N01 HC-55222, N01-HC-75150, N01-HC-45133, and N01-HC-85239; grant numbers U01 HL080295 and U01 HL130014 from the National Heart, Lung, and Blood Institute (NHLBI), and R01 AG-023629 from the National Institute on Aging, with additional contribution from the National Institute of Neurological Disorders and Stroke. A full list of principal CHS investigators and institutions can be found at https://chs-nhlbi.org/pi. This manuscript was not prepared in collaboration with CHS investigators and does not necessarily reflect the opinions or views of CHS, or the NHLBI. Support for the genotyping through the CARe Study was provided by NHLBI Contract N01-HC-65226.

The Framingham Heart Study (FHS) is conducted and supported by the National Heart, Lung, and Blood Institute (NHLBI) in collaboration with Boston University (Contract No. N01-

HC-25195 and HHSN268201500001I). This manuscript was not prepared in collaboration with investigators of the FHS and does not necessarily reflect the opinions or views of the FHS, Boston University, or NHLBI. Funding for CARe genotyping was provided by NHLBI Contract N01-HC-65226. Funding support for the Framingham Dementia dataset was provided by NIH/NIA grant R01 AG08122.

UK Biobank is a large-scale biomedical database and research resource containing genetic, lifestyle and health information from half a million UK participants. UK Biobank's database, which includes blood samples, heart and brain scans and genetic data of the 500,000 volunteer participants, is globally accessible to approved researchers who are undertaking health-related research that's in the public interest.

UK Biobank recruited 500,000 people aged between 40-69 years in 2006-2010 from across the UK. With their consent, they provided detailed information about their lifestyle, physical measures and had blood, urine and saliva samples collected and stored for future analysis.

UK Biobank's research resource is a major contributor in the advancement of modern medicine and treatment, enabling better understanding of the prevention, diagnosis and treatment of a wide range of serious and life-threatening illnesses – including cancer, heart diseases and stroke. Since the UK Biobank resource was opened for research use in April 2012, over 20,000 researchers from 90+ countries have been approved to use it and more than 2,000 peer-reviewed papers that used the resource have now been published.

UK Biobank is generously supported by its founding funders the Wellcome Trust and UK Medical Research Council, as well as the British Heart Foundation, Cancer Research UK, Department of Health, Northwest Regional Development Agency and Scottish Government. The organisation has over 150 dedicated members of staff, based in multiple locations across the UK.

This research has been conducted using data from UK Biobank, a major biomedical database.
